# Supplementary figures and images for: Functional and structural analyses of novel Smith-Kingsmore Syndrome-Associated MTOR variants reveal potential new mechanisms and predictors of pathogenicity
Source: PLoS Genet. 2021 Jul 1;17(7):e1009651. doi: 10.1371/journal.pgen.1009651 (PMC8279410; doi:10.1371/journal.pgen.1009651)

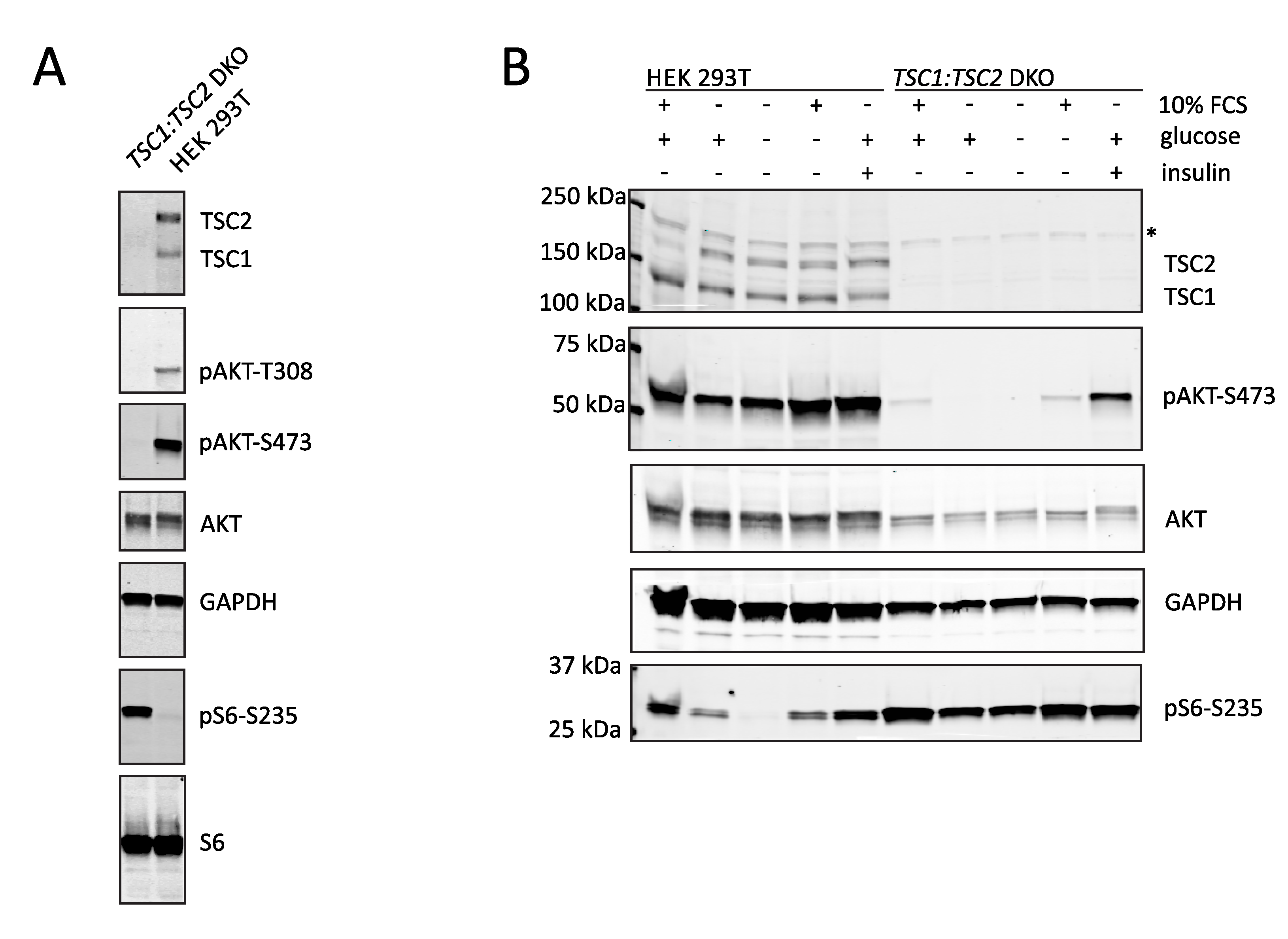

Supplement: S1 Fig — (A and B) 3H9-1B1 (HEK 293T TSC1:TSC2 DKO) cells exhibit reduced AKT activation. TSC1:TSC2 Double Knockout (DKO) cells were compared to the parental HEK 293T line by immunoblotting. Under conditions of nutrient and/or growth factor restriction, activated S473-phosphorylated AKT is down-regulated in the TSC1:TSC2 DKO cells, compared to the HEK 293T line. In contrast, under the same conditions, ribosomal protein S6, a marker for MTORC1 activation, is constitutively phosphorylated (pS6-S235) in the TSC1:TSC2 DKO cells. * = background band from non-specific binding. (TIF) [file pgen.1009651.s001.tif]

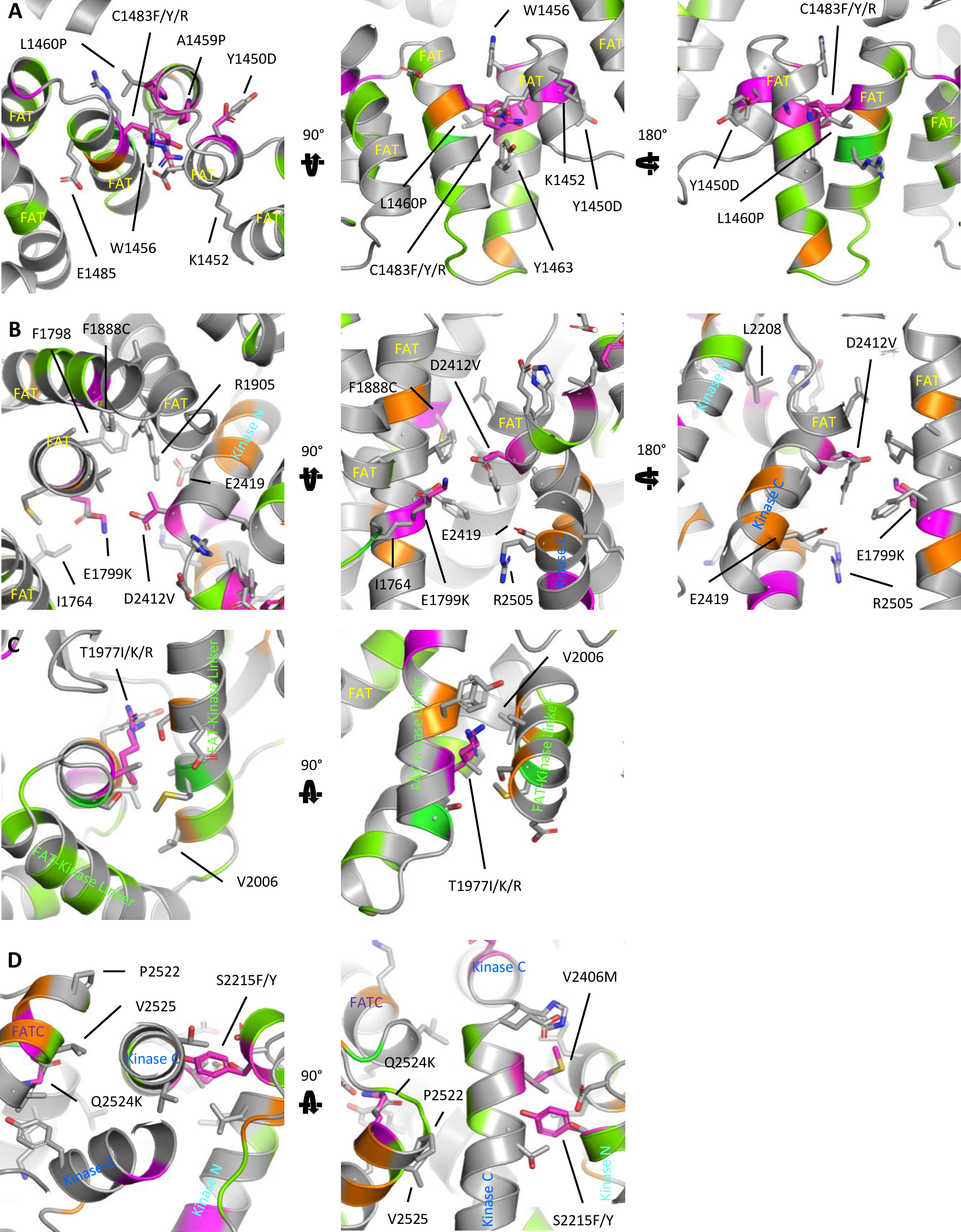

Supplement: S2 Fig — Detailed view of other mutation clusters associated with NDD phenotypes. (A) p.C1483 mutation cluster. (B) p.E1799 mutation cluster. C. p.T1977I/K/R. D. p.S2215F/Y. Wild-type side chains are shown as gray sticks, mutated side chains are shown as magenta sticks. Neighboring residues in close proximity are shown as gray sticks. Mutations associated with overgrowth phenotypes are shown in magenta, mutants from the COSMIC database in orange and SNPs from the gnomAD database in green. (TIF) [file pgen.1009651.s002.tif]

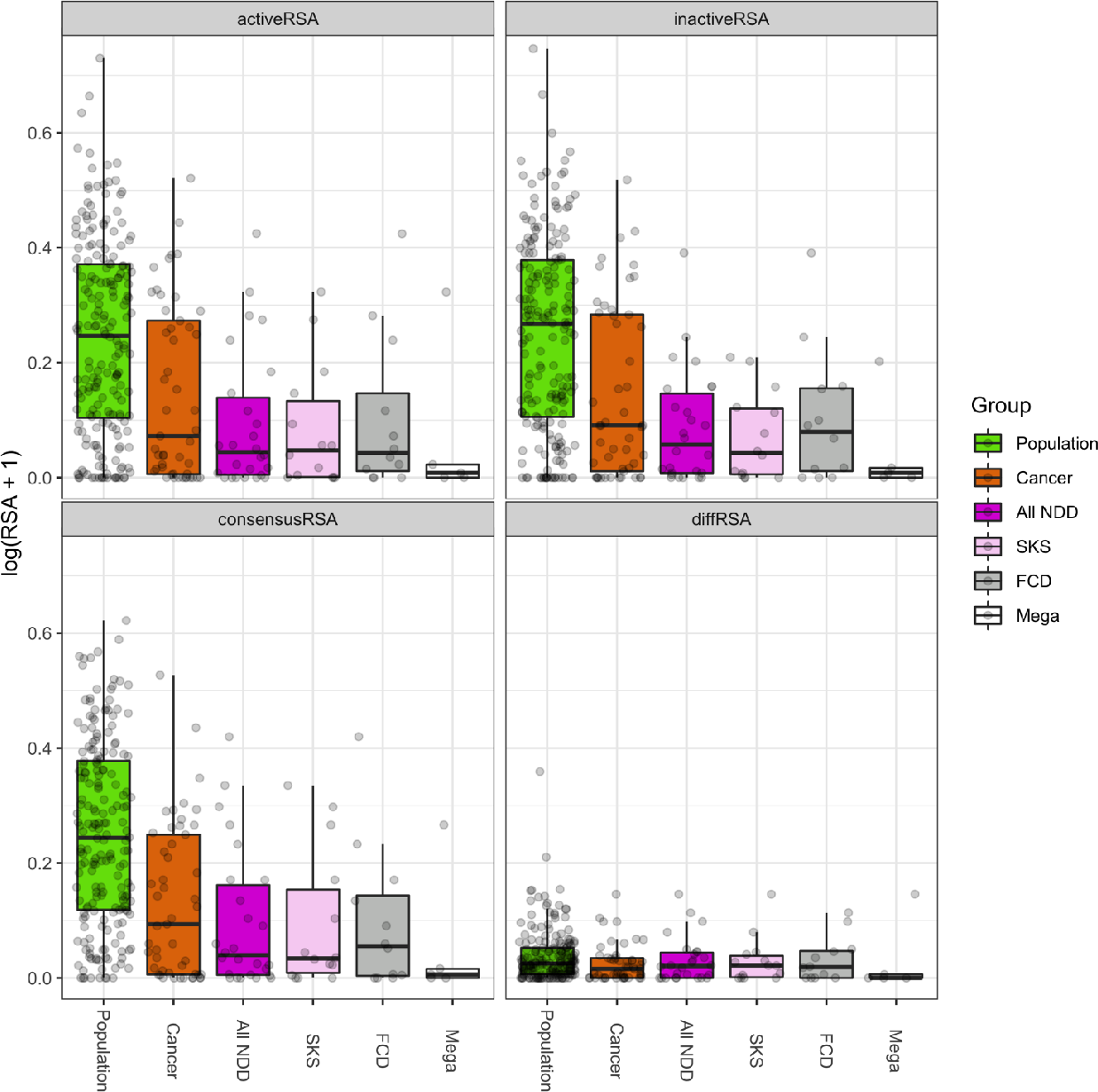

Supplement: S3 Fig — The gray panel headings indicate RSA values for the active, inactive, consensus of all structures, and differential between active and inactive, protein states. SKS, FCD and Mega are subgroups within the “All NDD” group. RSA = Relative Accessible Surface Area; NDD = Neurodevelopmental Disorder; SKS = Smith-Kingsmore Syndrome; FCD = Focal Cortical Dysplasia; Mega = Megalencephaly. (TIF) [file pgen.1009651.s003.tif]
