# Supplementary material for: Functional and structural analyses of novel Smith-Kingsmore Syndrome-Associated MTOR variants reveal potential new mechanisms and predictors of pathogenicity
Source: PLoS Genet. 2021 Jul 1;17(7):e1009651. doi: 10.1371/journal.pgen.1009651 (PMC8279410; doi:10.1371/journal.pgen.1009651)
Supplement: S1 Table — COSMIC = Catalogue of Somatic Mutations in Cancer; gnomAD = The Genome Aggregation Database; MTOR = mechanistic target of rapamycin; N/A = Not Applicable; SKS = Smith-Kingsmore Syndrome. (DOCX) [file pgen.1009651.s004.docx]

| **MTOR Variant** | **Neurodevelopmental Disorder Phenotype** | **Phenotype** | **Reference** | **Database** |
| --- | --- | --- | --- | --- |
| p.C1390Y | SKS | N/A | Novel, Current study | N/A |
| p.C1483F | SKS | Clear cell and renal cell carcinoma | [6] | COSMIC |
| p.E1799K | SKS | Many different cancers | [6] | COSMIC |
| p.V2406M | SKS | Astrocytoma, breast, kidney | Novel, Current study | N/A |
| p.V2406A | N/A | Clear Cell and Renal Cell Carcinoma | [16] | COSMIC |
| p.E2419K | N/A | Many different cancers | [17] | COSMIC |
| p.P2522A | N/A | N/A | N/A | gnomAD |
| p.Q2524L | N/A | Endometrium and Kidney Cancer | N/A | COSMIC |
| p.Q2524K | SKS | N/A | Novel, Current study | N/A |
| p.V2525I | N/A | N/A | N/A | gnomAD |
