# Supplementary material for: Functional and structural analyses of novel Smith-Kingsmore Syndrome-Associated MTOR variants reveal potential new mechanisms and predictors of pathogenicity
Source: PLoS Genet. 2021 Jul 1;17(7):e1009651. doi: 10.1371/journal.pgen.1009651 (PMC8279410; doi:10.1371/journal.pgen.1009651)
Supplement: Attachment — Submitted filename: plosgenetics.docx [file pgen.1009651.s008.docx]

This manuscript described novel clinical features of patients with SKS and found four pathogenic MTOR variants relevant with MTOR Complex 1/2 activation. The relative accessible surface area was significantly lower for SKS-associated variants

compared to benign variants. With some structural analysis, the author state that these MTOR variants may help in pathogenicity classification. Overall, this manuscript gives some findings for SKS disease, however there are some questions:

1. Figure 1 quality is too low and the enzyme assay is not so good to get a clear result. The S6K and AKT should be the same level if you transfect the same amount for each MTOR variant. It is weird that p-S6K level is increased without transfecting mTOR? How many times was this experiment repeated? At least, the figure 1A/E aren’t so convinced of the result.
2. Figure 2 A/B is almost the same, the quality is too low to see the detailed amino acids. Figure 2 and 3 are analyzed based on the previous structure.

The quality of this manuscript, especially the figures, are not good. There was no obvious novelty of this manuscript. Thus, it is not recommended to be published on the PLOS Genetics journal.
