# Supplementary material for: Functional and structural analyses of novel Smith-Kingsmore Syndrome-Associated MTOR variants reveal potential new mechanisms and predictors of pathogenicity
Source: PLoS Genet. 2021 Jul 1;17(7):e1009651. doi: 10.1371/journal.pgen.1009651 (PMC8279410; doi:10.1371/journal.pgen.1009651)
Supplement: Attachment — Submitted filename: Besterman_Response_to_Reviewers2.docx [file pgen.1009651.s009.docx]

**Reviewer #1:**

Authors report 7 patients with SKS with 4 novel MTOR variants, describe new phenotypes, confirm MTOR activation of the variants by detecting MTORC1 activation, and describe MTORC2 activation as a new potential mechanism in SKS. Authors found that pathogenic MTOR variants cluster in hotspots in the core of the protein and suggest that 3D properties of MTOR may help in the pathogenicity classification of the variants.

The description of new patients and variants and the functional confirmation of the activity of these variants are of great importance to increase the knowledge in this rare disease with very few cases described to date. Also, the three-dimensional approach as an extra element for assessing a variant's pathogenicity is quite exciting and promising.

However, there are a couple of things in the results that catch my attention. Below I write some questions and comments that arose when reviewing the work.

Minor:
ABSTRACT: Please check the verb tenses in the abstract.

We thank the reviewer for pointing this out. We have updated the verb tense from past tense to simple present tense in the abstract.

Lines 699-700: Please enter the text for 1G and 1H in alphabetical order, as it appears in the image, to avoid confusion.

We appreciate the reviewer pointing out this mistake. We have now corrected it so that they are in appropriate alphabetical order.

Lines 113-115: "Germline mosaic and constitutive mosaic (e.g., present in all cell types but at different frequencies) MTOR variants cause Smith-Kingsmore syndrome (SKS)." The term "germline mosaic" should be changed to "germline". If the authors want, it can also be mentioned that germline variants in SKS often originate from a "germline mosaicism" in one parent. On the other hand, the term "constitutive mosaic" is used in reference 3 to refer to a mosaic mutation present in all cell types but not in 100% of the cells. However, in a recent review and proposal to classify somatic mosaicism (in which many of the authors of reference 3 participate. See PMID: 32661356), it has been preferred to discontinue using the term "constitutional mosaicism" as it can be confusing because the term "constitutive" by itself is used to denote the opposite (non-mosaic variants). I suggest using "somatic mosaic" so that the whole sentence reads: "Germline and somatic mosaic MTOR pathogenic variants cause Smith-Kingsmore syndrome (SKS)".

We thank the authors for bringing up this important issue with terminology in reference to mosaicism, which can be very confusing. We updated our terminology to align with these recommendations and have added the suggested reference in order to inform readers as well.

Major:
In the Results section, the authors say that (lines 184-185) "Unexpectedly, the T389/S6K ratio in the absence of MTOR had an approximately 2-fold increase relative to wild-type MTOR". Later in the Discussion section, the authors say that (lines 298-300) "Unexpectedly, we found that exogenous expression of wild-type MTOR resulted in decreased MTORC1 activity". So, in the results section, the authors seem to assume that the construct without MTOR causes increased activity of MTORC1, while in the discussion section, the authors seem to assume the opposite, that is, it is actually the construct with the wild-type MTOR the one causing a decrease in MTORC1 activity. It is not very clear which of the two occurs, nor do the authors give a plausible explanation that justifies it. They do suggest, without explaining it, that (lines 300-301) "the exogenously expressed MTOR may interfere with the formation of active MTORC1 complexes". If this is so, could it be affecting the constructs with the mutations as well? Would this invalidate the results about MTORC1 activation? The same construct seems not to affect the MTORC2 pathway since the S473/AKT activity is similar with the exogenous wild type MTOR or with the plasmid without MTOR. Please explain.

We thank the reviewer for their comments on this important point. We now recognize that we need to update our terminology and elaborate on our explanation in order to make the interpretation of our functional results more clear. For the western blot figure, we have now changed the lane label from “-” to “Empty Vector (EV)” and “MTOR” to “Wildtype”. Throughout the text, and particularly in the result section describing these functional studies, we update our terminology so that we are now using the terms “empty vector” and “wildtype *MTOR*” to compare our two controls. With this new terminology and some additional explanation, hopefully it becomes clear that our two statements in our previous draft are not contradictory, but consistent with one another.

What we believe is occurring is that transfect a large quantity of wildtype MTOR, this activates a well-known MTORC1-dependent negative feedback loop, thus decreasing overall MTORC1 activity as measured by S6K phosphorylation. However, when we transfect a pathogenic MTOR variant that is endogenously activated due to the mutation, this activity seems to exceed the negative feedback loop activated by the higher quantity of MTOR transcript present, and thus you end-up with elevated MTORC1 activity overall. Note, that this feedback loop is independent of MTORC2, which is consistent with our observations, where we don’t see a reduction in S473/AKT with the transfection of wildtype MTOR. This has now all been clarified in both the results and discussion sections.

Lines 195-200: "Except for the p.C1483F variant, we did not observe large differences in the S473/AKT ratio. In the presence of the p.C1390Y and Q2524K variants the S473/AKT ratios were increased approximately 20%...In contrast, the S473/AKT ratios for the p.K1395R and p.V2406M variants were not significantly different from wild-type MTOR". If I have not misinterpreted, these do not appear to correspond to what is shown in figure 1, where (although with a larger variation bar) the p.V2406M variant appears to have a slightly higher ratio than the p.C1390Y variant. Please check to be able to assess these results.

We thank the reviewer for bringing our attention to this important issue. The reviewer is correct that S473/AKT for p.V2406M increases above WT by a factor of 1.27 and p.C1390 increases by a factor of 1.21. However, the mean S473/AKT for p.V2406M was calculated from 12 experimental replicates whereas the mean S473/AKT for p.C1390Y was calculated from 8 replicates, so we have less power to detect a difference from wildtype for p.C1390Y. This is reflected in our p-values, where the p-value for p.V2406M is 0.02 and for p.C1390Y is 0.06 (just shy of significant). The raw data has now been included as a supplement and can be viewed by the reviewer. We have reworded the paragraph in the results section to make this point a little clearer and it now reads as follows:

“To estimate the effects of the MTOR variants on MTORC2 activity, we expressed the variants together with an AKT3 reporter construct in 3H9-1B1 (TSC1:TSC2 KO) cells (Figures 2E - H). All transfected variants were expressed at approximately equal levels (Figures 2E and 1F). To compare the MTORC2 activity between variants, we determined the S473/AKT ratio in the presence of the transfected MTOR variants relative to transfected wild-type MTOR (Figures 2E and 2G). The p.C1483F variant, a previously reported highly activating variant [6,16] (Table 2), was associated with an approximate doubling of S473/AKT. The degree of activation of MTORC2 by the novel variants was more modest. S473/AKT increased approximately 1.5-fold compared to transfected wildtype for p.Q2524K and 1.2-fold for p.C1390Y and p.V2406M. Due to a larger number of replicates for p.C1390Y, it was found to be significantly greater than the transfected wildtype MTOR (p < 0.05, Student's paired t-test with Bonferroni correction), while p.V2406M was not (p = 0.06). The S473/AKT ratios for the p.K1395R and p.V2406M transfected variants were not significantly different from transfected wild-type MTOR.”

**Reviewer #2:**

This is nice manuscript focusing on the functional aspects of Smith-Kingsmore syndrome and the behavior of mTOR variant proteins.
The paper increases the knowledge of the biology of this important pathway and adds new useful information for the scientific community.
I have some suggestions to improve the MS:

1- Regarding the biology of the variant mTOR protein, more information of the potential treatments and experimental compounds should be incorporated in the text.

We thank the reviewer for this comment, as potential new therapies are vitally important to this patient population. We added the following sentence to the discussion proposing some potential therapeutic strategies that could be explored:

“Alternative treatment strategies may include AKT inhibitors that have shown promise in the related overgrowth disorder of Proteus Syndrome [43], dual MTORC1/2 inhibitors that are in development in oncology [44], or antisense oligonucleotide therapies directed towards *MTOR* transcripts[44].”

2- It would be important for clinicians to include clinical pictures of the patients’ phenotype.

We initially included photos of two patient in our resubmission, however, upon request of the PLOS Genetics editor, we have subsequently removed them. They stated to us: “We aim to avoid publishing identifiable images wherever possible, but even more so when the images are of children- in such cases, we would only allow the inclusion of images where absolutely essential and where no description of the clinical features could provide the necessary level of scientific understanding.” Please reach out to the editors with further questions/concerns about this issue.

3- The description of the CNS findings, mainly focused in the MRI/CT studies should be discussed in the text.

We thank the reviewer for their recommendation. We agree that a more extensive discussion of neuroimaging findings is warranted.

In the “Patient Demographics and Clinical Phenotypes” subsection of the Results, we added the following sentence: **“**Four of the patients had abnormal findings on neuroimaging, with pathology of the corpus collosum in two being the only commonality.”

In the Discussion section we added the following in reference to patient 7:

“While there were neuroimaging abnormalities in other patients, such as hemispheric asymmetry in patient 3 and thinning of the chiasma opticum in patient 6 (Table 1), there was no consistent pattern of abnormalities across all patients suggesting a specific progression of neurodegenerative changes. In fact these observations suggest significant heterogeneity in gross nervous system pathology in SKS.”

Minor comments:

The term “1” in number must be replaced by “one” (page 7, l.159 and page 15, l.289).

We thank the reviewer for pointing out these errors. We have made the suggested corrections.

**Reviewer #3:**

The authors identified 4 novel MTOR mutations from 7 patients sufficing SKS, and tested in vitro the MTOR variant expression levels and substrate phosphorylation ratios of MTORC1 and MTORC2. These parts are interesting and really impressive. However, the correlation between accessible surface area of mutated resides and the quantitative phosphorylation function is questionable. Mutations make effects by affecting the expression levels of the variants or by finely adjusting the relative orientation of N-lobe and C-lobe of kinase domain. Especially, the mutations within the helical interface of FAT domain may stretch or shrink the FAT helical bundles and therefore affect the kinase domain architecture. As a matter of fact, accessible surface area of 3D protein structure is a community effect of all amino acid side chain. Frankly speaking, it is a statistical quality meaningful only for the whole protein or entire interface. Anyway, although the authors have differentiated the RSA with the multi-sample nonparametric test, the linkage seems a little bit weak.

We appreciate the detailed feedback provided by the reviewer. By no means we intend to draw a direct correlation between accessible surface area and phosphorylation. Mutations can indeed have various effects on a protein, including expression levels. But as can be seen in figure 2, with few exceptions, expression levels of the mutants are similar or even slightly higher than wildtype protein. We agree with the reviewer, and also state this in the manuscript, the effects mutations on activity are most likely indirect, as also suggested by Yang et al. This can be either by disturbing the helix packing in the FAT domain and thereby loosening the inhibitory grip of the FAT domain around the kinase domain. Alternatively, they could also change the interactions of the kinase domains with each other and create a wider access to the catalytic cleft. A statement regarding the latter possibility has been included in the discussion.

However, we hope that the reviewer agrees with our conclusion that mutations in the core of the protein and closer to the active site can be expected to be more deleterious than those in the periphery. In this context accessible surface area, which can be calculated for individual residues in a structure, can serve as an, at least, semi-quantitative measure of the accessibility of a residue and therefore can, to an extent, serve to predict the severeness of mutation effects.

And while we would have preferred a stronger correlation, it cannot be ignored either. However, the number of known *MTOR* disease-associated mutations is currently still limited and more work on different proteins is certainly required as well.

A few comments:
1. Page 37, legend for Fig. 1. (H) & (G) should be exchanged.

We appreciate the reviewer pointing out this mistake. We have now corrected it so that they are in appropriate alphabetical order.

2. Page 37, legend for Fig. 2. 6BCX & 6BCU are cryo-EM structures, not crystal structures.

We appreciate the reviewer pointing out this mistake, we have now updated Figure 2 Legend to state: “Cryogenic Electron Microscopy Structure of MTOR with Mapped Novel Variants”

3. In the 3.0 A resolution cryo-EM map of 6BCX, the side chain assignment is only confident starting from L599 of N-HEAT domain. Therefore, any discussion about mutations before residue 599 of N-HEAT in Fig 4 is unbelievable.

We agree with the reviewer that the resolution of the cryo-EM map is a bit too low to confidently model all side chains and that parts of the structure are not fully resolved. However, all NDD mutations are located after L599, as are the majority of cancer-associated mutations. We have included a statement regarding the limitations of the structure at the current resolution.

4. Furthermore, the authors should not trust the coordinate model too much directly downloaded from Protein Data Bank, and should re-examine every important rotamer discussed in the cryo-EM map. For example, side chain of C1390 in Fig. 3A actually is in the same orientation as the mutated Y, if carefully inspected in the cryo-EM map.

As above, we agree with the reviewer on the limited resolution of the structure which makes modeling sidechains challenging. Furthermore, one always has to keep in mind that structures represent only a snapshot. For C1390 the map indeed seems to indicate a different rotamer than modeled. However, density for the sidechain of neighboring Q1355 is also largely missing. Hence alternative options for modeling rotamers in this area are possible. However, we don’t think that this changes much of the fact that the helices are very tightly packed and that replacing a small sidechain like cysteine with a larger/bulkier one like tyrosine disrupts this packing. We inspected other regions critical for our analysis and did not find other issues within the limitations of the map.

**Reviewer #4:**

This manuscript described novel clinical features of patients with SKS and found four pathogenic MTOR variants relevant with MTOR Complex 1/2 activation. The relative accessible surface area was significantly lower for SKS-associated variants
compared to benign variants. With some structural analysis, the author state that these MTOR variants may help in pathogenicity classification. Overall, this manuscript gives some findings for SKS disease, however there are some questions:

1. Figure 1 quality is too low and the enzyme assay is not so good to get a clear result. The S6K and AKT should be the same level if you transfect the same amount for each MTOR variant. It is weird that p-S6K level is increased without transfecting mTOR? How many times was this experiment repeated? At least, the figure 1A/E aren’t so convinced of the result.

We thank the reviewer for their close attention to these important issues. We agree that the quality of the figure in the PDF is too low for publication. This was due to figure compression requirements from the Journal on initial review. To be certain, we emailed PLOS Genetics editorial team who responded with the following:

“The compiled PDF compresses the figures to reduce the size of the file and make it easier for the Editor and reviewers to download. Please be assured that, should your manuscript be accepted for publication, our Production team will source the figures from the original files and not the PDF.”

We also emailed the PLOS production/graphics team who specifically reviewed our originally submitted figures and responded as follows:

“I pulled your figures from your manuscripts File Inventory and ran them through PACE and PACE ok'ed them all without making any changes. I visually inspected them and they look production ready, meaning we would not have to make any adjustments to your files. You created them correctly and the ones you submitted with your submission are ok as is…In the review PDF, figure images appear at lower resolution within the PDF, so it’s easier for editors and reviewers to download. However, you'll note that at the top of each figure page in the PDF, there’s a link to click and download the full figure file. If your figures appear correctly when you click to download the full resolution file, then there's nothing to be concerned about.”

Regarding the pS6K level, we realized that our previous wording needed additional clarification. In order to do so, we changed the “-“ label on the immunoblot to read “empty vector” and “MTOR” to read “Wildtype”. We also updated the wording throughout the text to reflect this as well. Hopefully, this new wording, along with additional explanation in the results and discussion sections, will help clarify the interpretation of these results, which is the following:

It is not that pS6K increased without transfecting MTOR, but that *pS6K decreased* when wildtype MTOR was transfected compared to cells transfected with an empty vector. We used the wildtype transfection as our comparator for all other variants (thus its value is set at 1). We think that transfecting high quantitates of wildtype MTOR activates a well-described MTORC1-dependent negative feedback loop that suppresses pS6K levels. For the NDD-associated variants, it seems that the endogenous protein activation caused by the mutations is able to overcome the effect of the negative feedback loop resulting in overall elevated pS6K.

Each immunoblotting experiment was repeated at least 5 times. We have now added that to our methods section in the “functional analysis” section. We thank the reviewer for highlighting that oversight.

2. Figure 2 A/B is almost the same, the quality is too low to see the detailed amino acids.

We thank the reviewer for their comments. As in reviewer 4, comment 1 above, we agree that the quality of the images presented to the reviewers in the PDF is too low and make it difficult to distinguish the details of the amino acids and thus the difference between figures 2A/B. This was due to figure compression requirements from the Journal on initial submission. We will certainly work with the Journal to provide high resolution, high quality images for final publication so that all necessary details of the amino acids can be visualized properly and have verified with Journal staff that this will be feasible.

Figure 2 and 3 are analyzed based on the previous structure.

We thank the reviewer for pointing this out and expressing their concern. We decided to use publicly available protein structures for our analysis as the main focus of our research study was to understand the impact of novel variants on *MTOR* structure and function and less on developing newly refined structures, which was outside the scope of this study. 6BCX, the structure we used in this study, is the latest structure of MTORC1 with the highest resolution. Later structures of MTORC1 have significant lower resolution. 6ZWO is more recent, however this structure is of MTORC2 and was released after completion our analysis.

The quality of this manuscript, especially the figures, are not good.

Please see our above response to reviewer #4, question 2.

There was no obvious novelty of this manuscript. Thus, it is not recommended to be published on the PLOS Genetics journal.

We thank the reviewer for their concern. Regarding novelty, our work makes several novel and important contributions to the understanding of SKS, a rare and understudied genetic disorder. As reviewers 1 and 2 highlight, we describe 4 novel variants that will help make future diagnoses of SKS easier. We also report new clinical findings and expand the known phenotype of SKS. We confirm that MTORC1 activation is the primary mechanism stimulating cellular overgrowth and provide evidence that MTORC2 activation may be a secondary, lesser mechanism of growth stimulation for some variants. This finding has clinical implications, suggesting that therapeutics should probably target both pathways instead of MTORC1 only. We also provide evidence that including the 3D protein impact of variants on MTOR in pathogenicity classification may be helpful. We feel strongly that our work makes meaningful and novel contributions to the field and to our patients.

Reviewer #1: **No:** The data associated with the Figures has not been provided

We thank the reviewer for pointing this out. We have now added supplemental spreadsheets with the data used to create Figures 2 (AKT_SUP_DATA; S6K_SUP_DATA), 5 (RSA_SUP_DATA), and 6 (RSA_SUP_DATA). There is no data to accompany Figures 1, 3, and 4.
